# Expression analyses of chemosensory genes provide insights into evolution of gustatory receptor genes in the bumble bee *Bombus impatiens*

**DOI:** 10.1186/s12864-025-11710-x

**Published:** 2025-07-01

**Authors:** Kaleigh Fisher, Blanca M. Guillén, Anupama Dahanukar, Naoki Yamanaka, S. Hollis Woodard

**Affiliations:** 1 Department of Entomology, University of California, Riverside, USA; 2 Department of Evolution and Ecology, University of California, Davis, USA; 3 Department of Molecular, Cell and Systems Biology, University of California, Davis, USA

**Keywords:** Chemoreceptors, Bumble bees, Gene duplications, Tissue-specificity

## Abstract

**Background:**

The chemoreceptor gene families, which include gustatory, ionotropic, and odorant receptor gene families, are highly conserved across the insects yet also contain many recently duplicated, species-specific genes in particular clades. Within the Bombini (bumble bee) clade, a set of recently duplicated genes in the gustatory receptor (*Gr*) gene family was identified, the functions of which are unknown. Recently duplicated genes are hypothesized to first evolve to serve non-essential functions, then subsequently evolve additional functions. We tested support for this hypothesis in the common eastern bumble bee, *Bombus impatiens*, by identifying recently duplicated *Gr* genes in this lineage and examining their tissue and caste expression patterns.

**Results:**

We annotated twenty-one *Gr* genes in *B. impatiens*. Ten of these genes appear to be unique to bumble bees and more likely to be expressed in a subset of sensory organs compared to the conserved *Gr* genes, which were more broadly expressed across sensory organs. This finding provides support for the hypothesis that younger genes have narrower functions and subsequently become more generalized over time.

**Conclusions:**

Recently duplicated genes can mediate behavioral and ecological shifts, making them intricately linked to evolutionary processes such as adaptation and diversification. Chemoreception mediates fundamental behaviors in bumble bees like finding mates, selecting suitable nest sites, and foraging resources. Despite this, the molecular basis of bumble bee chemoreception, and the importance of each chemoreceptor family to bumble bee behavior and ecology, are still relatively uncharacterized. This study helps to characterize how chemoreception evolves in bumble bees and provides a foundation for understanding what tissues are important for bumble bee chemoreception.

**Supplementary Information:**

The online version contains supplementary material available at 10.1186/s12864-025-11710-x.

## Background

Recently duplicated genes can mediate evolutionary processes such as adaptation and speciation because they provide new genetic material for natural selection to act upon [1]. They are generally species-specific and evolve via duplication and divergence events of protein-coding genes. After they duplicate, these paralogous genes can be quickly integrated into fundamental physiological processes [2].

Identifying recently duplicated genes and their functions is, therefore, intricately linked with understanding how new phenotypic traits arise and how organisms successfully colonize new environments. Truly species-specific genes can only be identified by comparing genes across the complete genomes of closely related species. The rapidly increasing availability of complete genomes for a broad range of species has led to the identification of many species-specific genes and subsequently raised many exciting questions about their evolution and functional significance.

Insect chemoreceptor gene families are generally highly conserved, but also contain many species-specific genes, with substantial differences in sequence and number even among closely related species [3–5]. This pattern is a result of these gene families evolving via birth-death evolutionary processes, which include gene duplication, loss, and pseudogenization events (Nei et al., 2008). Insect chemoreceptor genes share similar evolutionary histories and all belong to a superfamily that includes the gustatory receptor (*Gr*) gene family, the ionotropic receptor (*Ir*) gene family, and the odorant receptor (*Or*) gene family. The *Gr* gene family is the most ancient of the chemoreceptor gene families, followed by the *Ir* gene family [6]. The *Or* gene family evolved from the *Gr* gene family and appear to only be present in hexapods [7]. The putative functions of chemoreceptor genes in non-model organisms are typically inferred from orthology with genes in model species like *Drosophila melanogaster*, where more chemoreceptor genes have been de-orphanized (i.e., chemical ligands that bind to receptors have been identified) [8, 9]. For many chemoreceptor genes, particularly species-specific genes lacking orthologs with better-characterized species, it remains challenging to connect molecular changes in chemoreceptor genes to evolutionary, behavioral, and ecological changes because the basic functions of these genes are not known.

In bumble bees (genus *Bombus*, family Apidae within Anthophila), a group of economically important, generalist, eusocial pollinators, chemosensation plays important roles in mediating plant-pollinator interactions and social interactions within the nest. For example, chemosensation helps bumble bees discriminate between nutrients and toxins in floral resources during foraging bouts, and thus shapes which floral resources bees collect pollen and nectar from [10–12]. Chemosensation also plays an important role in conspecific communication, including pheromone-based mate location, nestmate recognition, and signaling of social status, for example through queen pheromones [13].

Within the Bombini clade (the lineage containing the bumble bee genus, *Bombus*), a set of recently duplicated genes in the *Gr* gene family was identified, which occurred in three separate (paralogous) gene expansions [14]. None of these genes is shared with the more closely-related western honey bee, *Apis mellifera* (family Apidae; estimated divergence time from bumble bees = 50–70 million years ago or MYA) [14–17], nor more distantly related bee species such as the alfalfa leafcutter bee *Megachile rotundata* (family Megachilidae; estimated divergence time = 75–100 MYA) [16]. One of these expansions in Bombini appears to share a common ancestor with a sister expansion in the stingless bee *Melipona quadrifasciata.* Stingless bees (Meliponini) are the sister group to Bombini, with the two lineages diverging at estimated 30–40 MYA [15]. The genes within each of these two clades’ expansions, however, do not appear to be paralogs [18], and thus they have independently diverged in the Bombini and Meliponi following their origin in the lineage leading to these two clades. All three *Gr* gene expansions appear to have evolved from genes that encode bitter-sensing receptors, based on orthology with *D. melanogaster* bitter Grs [14, 16, 19]. Beyond this, the specific compounds that any of these recently duplicated *Gr*s across the three bumble bee-specific expansions are involved in detecting, and the behaviors that they subsequently mediate, are completely unknown.

In this study, our primary goal was to examine the species-specific *Gr* genes in the bumble bee *Bombus impatiens* in more detail, with the main objective of exploring their patterns of expression as a foundational step towards understanding what their functions might be. With respect to their function, species-specific genes are hypothesized to first evolve to serve non-essential functions, and then subsequently evolve additional functions. A molecular prediction of this hypothesis is that species-specific genes are more likely to exhibit tissue-specific patterns of expression than conserved genes. This idea is supported by findings from *D. melanogaster* that newer genes tend to have a more restricted tissue expression range and evolve greater expression breadth over evolutionary time [20, 21]. In *B. impatiens*, based on the hypothesis that recently duplicated genes have fewer essential functions, we predicted that the species-specific *Gr*s would also have more tissue-specific patterns of expression, compared to more conserved and canonical receptors like the sweet receptors. To test these predictions, we first identified and annotated all the chemoreceptor genes (*Gr*s, *Ir*s, and *Or*s) in *B. impatiens*, and established which genes were species-specific in bumble bees based on comparisons between two species in Bombini, *B. impatiens* and *B. terrestris*, along with *D. melanogaster*, *A. mellifera*, and *M. quadrifasicata*. We then characterized chemoreceptor gene expression patterns across five tissues (antennae, brain, fat body, tarsi, and mouthparts) and in both female castes (workers and queens) to test the specificity of their expression across the contexts of tissue and social castes.

## Methods

### Bumble bee rearing

Three queen-producing and three worker-producing *B. impatiens* colonies were supplied by Koppert Biological Systems (Howell, MI). Twenty newly-eclosed (adult age < 24 h, or “callow”) individuals were selected from each colony (three queen-producing colonies; three worker-producing colonies) and placed into rearing cages, supplied by Biobest USA, Inc. (Romulus, MI, USA) in colony groups. Callow individuals were identified by their silvery appearance. Seven days after eclosion and placement in rearing cages, bees were frozen at -80 °C and stored at this temperature until tissue dissections. These methods allowed us to collect individuals that were age-matched (adult age = seven days) and collected at the same time of day to minimize age- and circadian-related differences in gene expression. All colonies and bees were maintained in either Biobest queen cages, or Koppert colony boxes, in groups of at least five bees. All bees were maintained under standard rearing conditions (25 ± 3 °C, 40 ± 5% relative humidity) and supplied with honey bee-collected pollen (stored frozen; obtained from Biobest USA, Inc. Romulus, MI) and artificial nectar syrup [22], both provided *ad libitum*.

### Sample preparation, RNA isolation, and sequencing

The following tissues were dissected over dry ice: (i) proboscises, which included the glossa, labial palps, and maxillary palps; (ii) antennae; (iii) front tarsi; (iv) brain; and (v) fat body. Proboscises were cut at the base of the clypeus. Both antennae were removed at the base of the head capsule and combined. Front tarsi were removed from the tibia and combined. Brains were removed from the head capsule through the dorsal side and the subesophageal ganglion was removed. Fat bodies were obtained from abdomens and were left connected to the abdominal cuticle [23]. For each tissue type, tissues from twenty individuals from the same source colony were pooled and stored in Trizol at -80 °C until RNA extraction; this equated to a total of three pools per tissue type, for each of the two castes. Tissues were homogenized in Trizol with two metal beads at maximum frequency for 5 min using a TissueLyser II (Qiagen). RNA was isolated from homogenized tissues using the RNeasy Mini Kit (Qiagen) according to the manufacturer’s protocol. Per sample RNA quality and quantity were assessed using agarose gel electrophoresis, nanodrop, and an Agilent 2100 bioanalyzer. RNA sequencing libraries were generated from 800 ng of total RNA per sample using Illumina’s TruSeq Stranded mRNA Sample Prep Kit following the manufacturer’s instructions. RNA libraries were multiplexed and sequenced with 150-bp paired-end reads (PE150) to a depth of ~ 20 million reads per sample on an Illumina HiSeq 4,000 at the Novogene Corporation Labs at UC Davis.

### Gene annotation

Although many chemoreceptor genes and isoforms are identified as such in the *B. impatiens* refseq assembly, the majority of chemoreceptor genes are not categorized as chemoreceptor genes in the assembly. To identify chemoreceptor genes that have already been identified as genes or isoforms, and find genes that were not previously identified as genes in the NCBI annotation pipeline, we used the bitacora gene family annotation pipeline (v 1.4) to manually annotate chemoreceptor genes in *B. impatiens* [24]. For each gene family, we used predicted protein sequences from *B. terrestris* as queries combined with HMM profiles for each gene family to identify predicted genes in the *Bombus impatiens* genome based on sequence similarity (TBLASTN) and gene family protein domains (e-value < 1e-3). Predicted genes were matched with previously annotated genes in the most recent *B. impatiens* refseq assembly (GCF_000188095.3). Genes not included in the annotation file were also generated using GeMoMa software from the TBLASTN alignments. Specific parameters for each gene family are included in the supplementary material. We manually inspected genes predicted by bitacora in Integrative Genomics Viewer (IGV) to confirm splice sites and confirm isoforms from the NCBI annotation file. We also inspected whether chemoreceptor domains were present and intact using NCBI’s Conserved Domain Search (https://www.ncbi.nlm.nih.gov/Structure/cdd/wrpsb.cgi*).Ir*s evolved from the ionotropic glutamate receptors [25] and can be distinguished based on presence of specific domains. We filtered 13 genes that had the presence of Kainate, AMPA or NMDA domains, which shared similar sequences to *Ir25a*. We added the manually curated novel gene annotations to the refseq GFF file using the agat_sp_merge_annotations.pl script from AGAT (https://github.com/NBISweden/AGAT). We then used the AGAT function to format the annotation file for downstream pipelines. We aligned the *B. impatiens* gene sequences to the previously annotated *B. terrestris* genes using MAFFT v7.520. We then named the *B. impatiens* genes based on sequence similarity to numbers in *B. terrestris* and otherwise attempted to follow naming guidelines from [26]. Additionally, we manually inspected the final gene annotations with the transcriptome data. Specifically, we merged and indexed bam files for the mouthpart, tarsi, and antennal transcriptomes for both workers and queens to visualize in IGV alongside predicted gene annotations.

### Phylogenetic analysis

For each chemoreceptor gene family, we aligned gene models from *B. impatiens* using the program MAFFT v7.520 (L-INS-I algorithm for one conserved domain: *Gr* and *Or* gene families; E-INS-I algorithm for more than one conserved domain: *Ir* gene family). We then built phylogenetic trees based on DNA sequences using IQ-TREE by combining the ModelFinder, tree search, SH-aLRT test and 1000 bootstrap replicates to select the best tree model [27]. Trees were plotted using FigTree v1.4.4. We also built a phylogenetic tree using the same methods that included Gr gene models for *B. impatiens*, *B. terrestris*, *A. mellifera*, *M. quadrifasciata*, and *D. melanogaster* to identify and visualize novel *Gr* genes. We rooted all trees at the nodes with the most conserved genes in FigTree v1.4.4 (e.g., genes that are present in all insects) following previous phylogenetic studies [19, 25]. These genes are *Or*: *Orco*; *Gr*: *Gr1* and *Gr2*; *Ir*: *Ir25a* and *Ir8a*. Tree output files for each tree, which include the models selected by ModelFinder and the bootstrap support values for each branch, are available in Supplementary File 5.

### Transcriptome assembly and differential gene expression analysis

Raw reads were trimmed using trim_galore v.0.6.6. The BIMP2.2 genome (GCA_000188095.4) was indexed with the most recent annotation (GCF_000188095.3) using STAR v2.7.1a with a 149 bp overhang. Reads were then aligned to the genome twice and sorted by genome coordinates. We then used featurecounts v. 2.0.6 to quantify reads. Specific parameters for each run can be found on OSF. We used the package *Deseq2* [28].

We used median of ratios to generate normalization factors of gene expression for each sample to account for differences in sequencing depth and RNA composition between samples. Genes with zero counts across all samples, extreme expression outliers, and low mean normalized counts were filtered from the dataset. Normalized counts of the complete dataset were then regularized log (*rlog*) transformed to moderate the variance across the mean. We then constructed a generalized linear model (glm), with caste, tissue, and the interaction between caste and tissue, as factors. We assessed sample quality and evaluated whether variance between samples could be explained by any of the factors in the glm, or natal colony, using the top 500 most variable genes in the dataset, to inform which factors should be included in the final generalized linear model. For this we used a principal component analysis (PCA) and hierarchical clustering analysis. Based on our quality control analysis, which demonstrated that variance between samples could be explained by caste and tissue, but not natal colony, we tested the effect of caste, tissue, and the interaction between caste and tissue on gene expression using a glm with a negative binomial distribution.

### Comparisons of chemoreceptor expression patterns across samples

We performed Wald tests using every gene in the complete dataset, with a Benjamini-Hochberg FDR correction (*p* < 0.05) to account for multiple comparisons. Results were then filtered to only include annotated chemoreceptor genes to test whether chemoreceptor gene expression differed between tissues and castes. Normalized counts of chemoreceptor genes were then used to visualize expression differences between genes and to visualize similarity in chemoreceptor expression between tissues and castes using a non-metric multidimensional scaling analysis (NMDS) with a Bray-Curtis dissimilarity index.

### Tissue specificity of gene expression

We used the normalized counts to quantify the tissue specificity of each gene by calculating the τ index for each gene [29] (https://github.com/severinEvo/gene_expression/blob/master/tau.R*).* The τ index ranges from 0 to 1, with 0 being no specificity to expression and 1 being fully tissue specific. We used a cut-off of 0.8 for a gene to be considered tissue specific. We performed a Fisher’s exact test to test whether it was more likely that the novel genes in *B. impatiens* (e.g., genes that do not share homologs with non-*Bombus* species) have tissue-specific expression. We considered a gene species-specific if it did not share a homolog with *M. quadrifasciata* or *A. mellifera*, the majority of which were in the three expansions.

## Results

### Identification and annotation of species-specific Gr genes

**Fig. 1:**
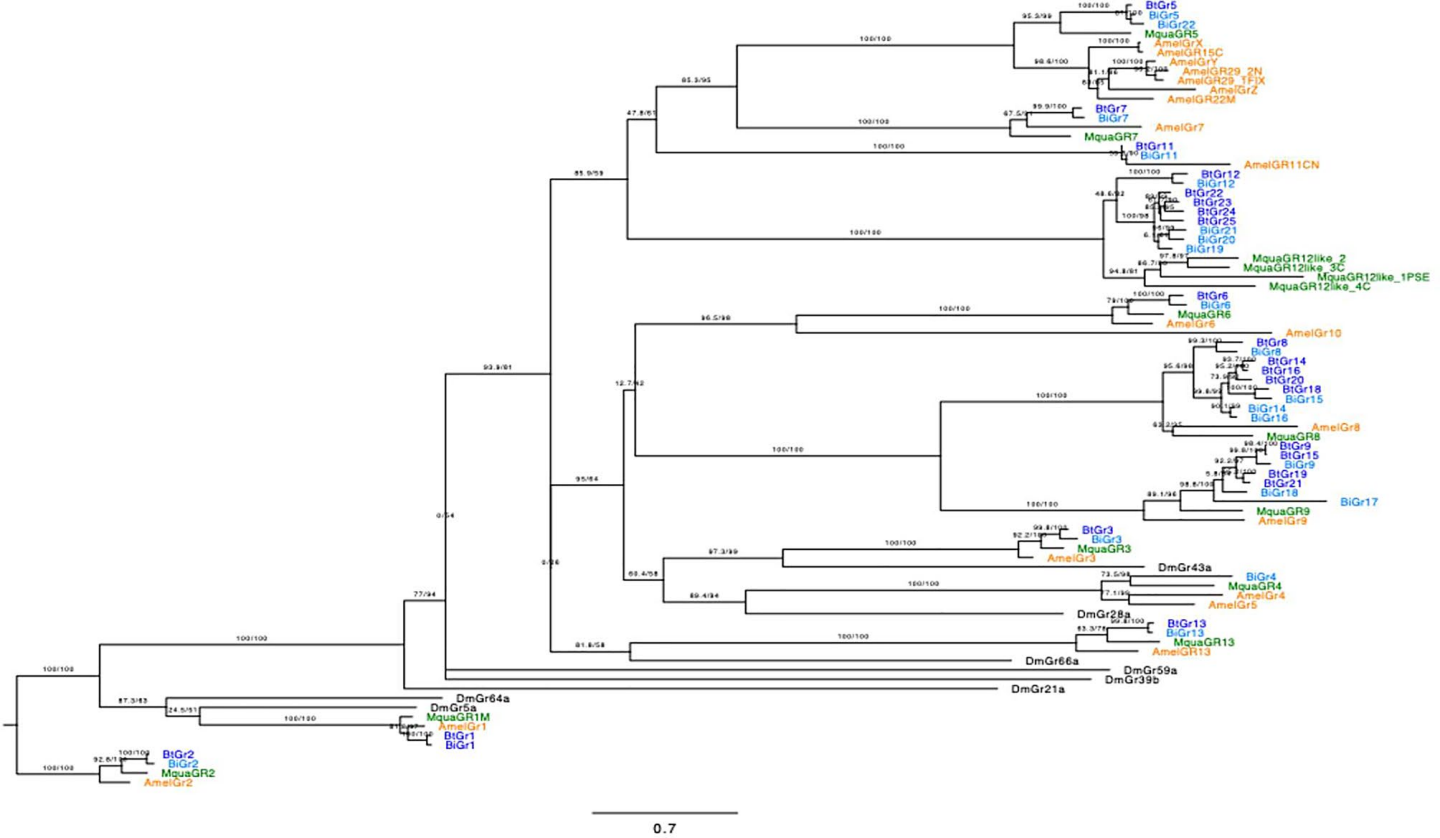
Gene tree with phylogenetic relationships between gustatory receptors in ***B***. *impatiens* (light blue), ***B***. *terrestris* (dark blue [14]), *M. quadrifasciata* (green [18]), ***A***. *mellifera* (orange; [19, 38]), and ***D***. *melanogaster* (black; [39]). Only a subset of ***D***. *melanogaster* Grs are displayed on the tree that share sequence similarity with bee Grs. Branch values indicate SH-aLRT support (%) / ultrafast bootstrap support (%). The substitution model selected by ModelFinder was Q.mammal + F + I + R4

We annotated 21 *Gr* genes, 23 *Ir* genes, and 158 *Or* genes in the *B. impatiens* reference genome, along with unaligned *B. impatiens* tissue transcriptomes generated in this study. These numbers only reflect putatively functional genes, as we did not include pseudogenes in our annotation. We considered genes to be pseudogenes if they had premature stop codons or missing start codons, following guidelines from [26]. Among the *Grs*, we identified ten species-specific genes, nine of which occurred in three putatively Bombini-specific *Gr* expansions in *B. impatiens* (Fig. 1). These gene expansions were also identified in *B. terrestris* [14], but not in other bee genomes (*A. mellifera*, *M. quadrifasciata*, *M. rotundata*) that have been considered in this study and in [16], which includes one member of the sister-lineage to bumble bees (*M. quadrifasciata* within the Meliponini). The tenth species-specific gene, *BiGr22*, is a duplication of *BiGr5*, which shares homologs with *B. terrestris* and *M. quadrifasciata*. The four genes in one expansion (*BiGr12*, *BiGr19*, *BiGr20*, and *BiGr21*), which also appears to have evolved from an ancestral gene that is shared with a sister expansion in *M. quadrifasciata* (Fig. 1), are all located on the same scaffold. There are three genes in a different expansion (*BiGr9*, *BiGr17*, and *BiGr18*) and four genes in another expansion (*BiGr8*, *BiGr14*, *BiGr15*, and *BiGr16*). With the exception of *BiGr16*, all genes in these latter two expansions are distributed between two scaffolds that are located close together in the genome. Two genes in the *Gr* gene family, *BiGr20* and *BiGr4*, have an incomplete N-terminus domain but otherwise appear to be intact and maintain high gene expression levels across various tissues. Six genes (*BiGr9*, *BiGr13*, *BiGr14*, *BiGr15*, *BiGr17*, and *BiGr22*) are missing the gustatory receptor domain (pfam08395). All these genes have relatively high expression levels across multiple tissues, with the exception *BiGr22*, which has lower expression levels but also appears to be an intact gene.

We annotated conserved *Gr* genes, including three that share orthologs with most other insects and are found in the other bumble bee with a fully sequenced genome, *B. terrestris: BiGr1*, *BiGr2*, and *BiGr3* (Fig. 1). *BiGr1* and *BiGr2* have homologs in *M. quadrifasciata*, *A. mellifera*, and *D. melanogaster*. They have been functionally characterized as sweet receptors in *D. melanogaster* [14, 19, 30, 31], and appear to be involved in sugar detection in bumble bees based on cellular recordings of these receptors [32, 33] and honey bees based on in vivo recordings and heterologous expression of these receptors [34]. *BiGr3* appears to be conserved across most insects [16] and has been functionally characterized as the fructose receptor in *D. melanogaster* [35] (Supplementary File 1: Bimp_Grs).

The *Ir* family in *B. impatiens* includes *BiIr8a* and *BiIr25a*, which are both highly conserved across insects [25]. *B. impatiens* has the same duplication of *Ir25a* that was found in *B. terrestris* [14]. We labeled the two genes *BiIr25a1* and *BiIr25a2*, consistent with [14] *B. impatiens* also shares the same antennal *Ir*s as *B. terrestris*: *BiIr93a*, *BiIr76b*, and *BiIr68a*, which are found in numerous other insects [25]. *Ir76b* has two isoforms, which we labeled as *BiIr76.a* and *BiIr76.b*. The remaining 18 genes are classified as divergent *Ir*s, consistent with [25]. Two of the divergent *Ir*s have two isoforms, which we distinguished with a and b: *BiIr334.a*; *BiIr334.b* and *BiIr75u.a*; *BiIr75u.b*. We included domain information for the *Ir*s in Supplementary File 1: Bimp_Irs.

The *Or* family includes *Orco*, the odorant receptor co-receptor that is broadly expressed in *D. melanogaster* olfactory receptor neurons and highly conserved across insects [16, 36]. *Orco* is the only shared *Or* between bees and *D. melanogaster* [14, 19]. We identified a large tandem duplication of *BiOr1*-*BiOr47* that is distributed across two closely located scaffolds (Supplementary File 1: Bimp_Ors), similar to the tandem duplications found in *(A) mellifera* and *(B) terrestris* [14]. *BiOr5* is homologous to *AmOr11*, which was characterized as the queen pheromone receptor in *(A) mellifera* [37]. The majority of *Or*s were intact with complete domains (Supplementary File 1: Bimp_Ors). The *Or* phylogenetic relationships are consistent with those found in *(B) terrestris* [14].

### Species-specific Gr genes are more likely to exhibit tissue-specific expression compared to conserved Gr genes

**Fig. 2:**
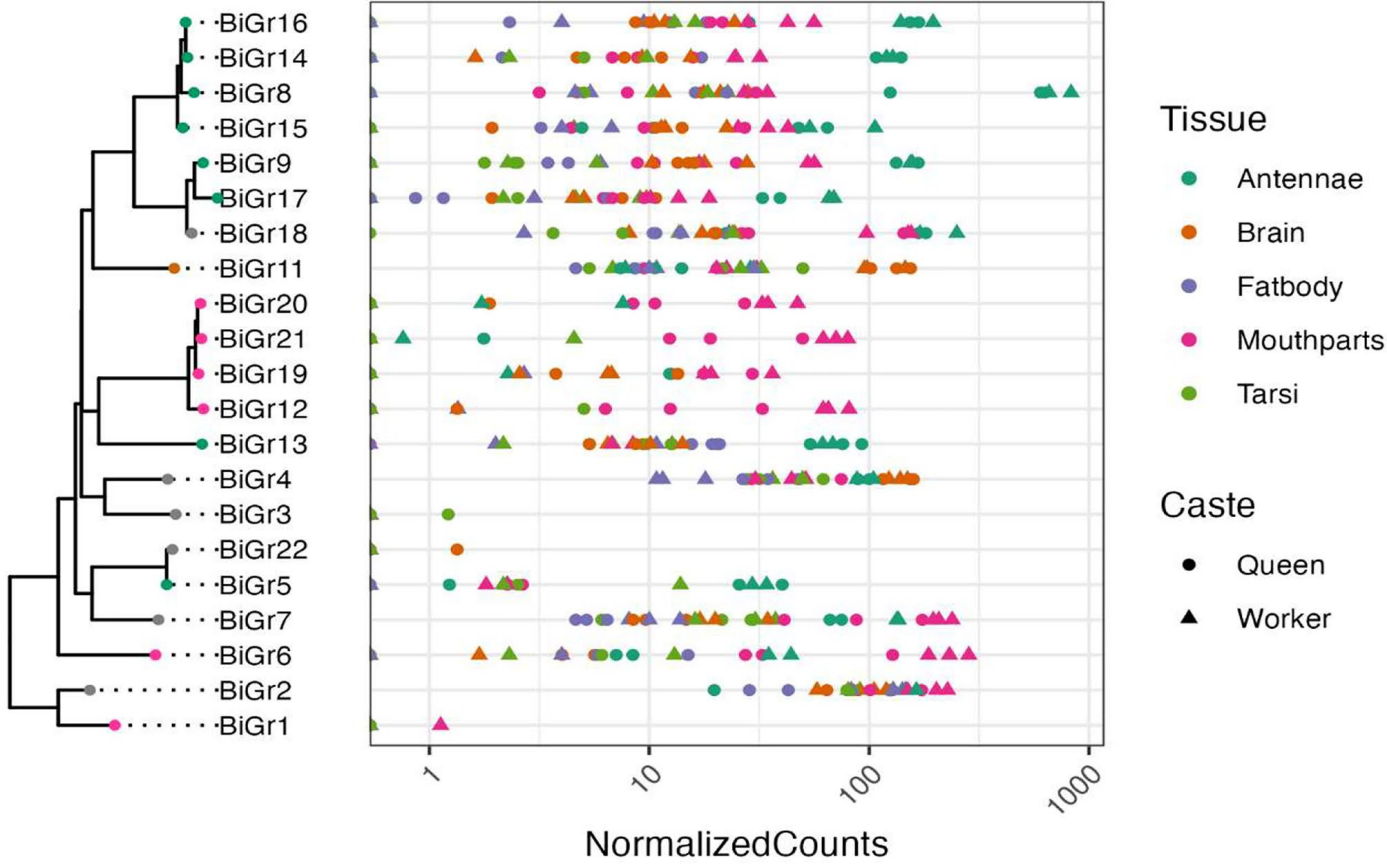
Gene tree with phylogenetic relationships between gustatory receptors. Colored circles on nodes of the phylogeny indicate the tissue that each gene is primarily expressed in (τ > 0.8) for the worker caste (queen tissue specificity is not shown). Grey circles on gene tree indicate genes that were not primarily expressed in any tissue (τ < 0.8). Normalized counts of gene expression for each gene represented on the gene tree for each tissue and caste are shown in the graph. The substitution model selected by ModelFinder was JTT + F + I + R3

We examined tissue specificity of expression for all chemoreceptor genes by comparing expression in the mouthparts, tarsi, antennae, brain, and fat body because mouthparts, tarsi and antennae are major chemosensory tissues in insects [40]. We included brain and fat body to compare internal and external nutrient sensing organs. Expression of chemoreceptors have previously been detected in these tissues in other insects [23;35]. Species-specific *Gr* genes were more often expressed specifically in one tissue compared to conserved genes (Fisher’s Exact Test: *p* < 0.05). *Gr* gene expression was most specific (τ > 0.8) to the antennae, followed by mouthparts, for both queens and workers (Figs. 2; S1). 15 out of 21 *Gr* genes in workers, and 16 out of 21 *Gr* genes in queens, were expressed specifically in a single tissue.

All four genes in the first *Gr* expansion (*BiGr12*, *BiGr19*, *BiGr20*, and *BiGr21*) were expressed specifically in the mouthparts of both queens and workers. As these four genes share sequence similarity and are located close together in the genome, they likely duplicated from a single ancestral gene and share a similar function in the mouthparts. The seven genes in the two additional expansions (*BiGr9*, *BiGr17*, and *BiGr18*; *BiGr8*, *BiGr14*, *BiGr15*, and *BiGr16*) are all expressed specifically in the antennae of both queens and workers. Similar to those in the *BiGr12* expansion, all of these genes that share sequence similarity are located closely in the genome.

Genes with tissue-specific expression exhibited consistent patterns between queens and workers, with three exceptions: *BiGr1*, a putative sugar receptor, was specific to mouthparts in workers but not specific to any of the tested tissues in queens; *BiGr3*, the putative fructose receptor, was specific to tarsi in queens but not specific to any tissue in workers; and *BiGr22* was specific to the brain in queens but not specific to any tissue in workers. All three of these genes, however, had low expression levels and it is possible that the observed specificity is an artifact of low expression rather than true restriction of expression.

*BiGr2*, a putative sugar receptor, and *BiGr4*, were both broadly expressed across tissues in queens and workers. *BiGr7* and *BiGr18* were not specific to any tissue, but both had higher expression levels in the mouthparts and antennae relative to other tissues (Fig. 2; Supplementary File 2; FDR-corrected *p* < 0.05). Most *Gr*s had the highest expression levels in the antennae and mouthparts (Fig. 2).

**Fig. 3:**
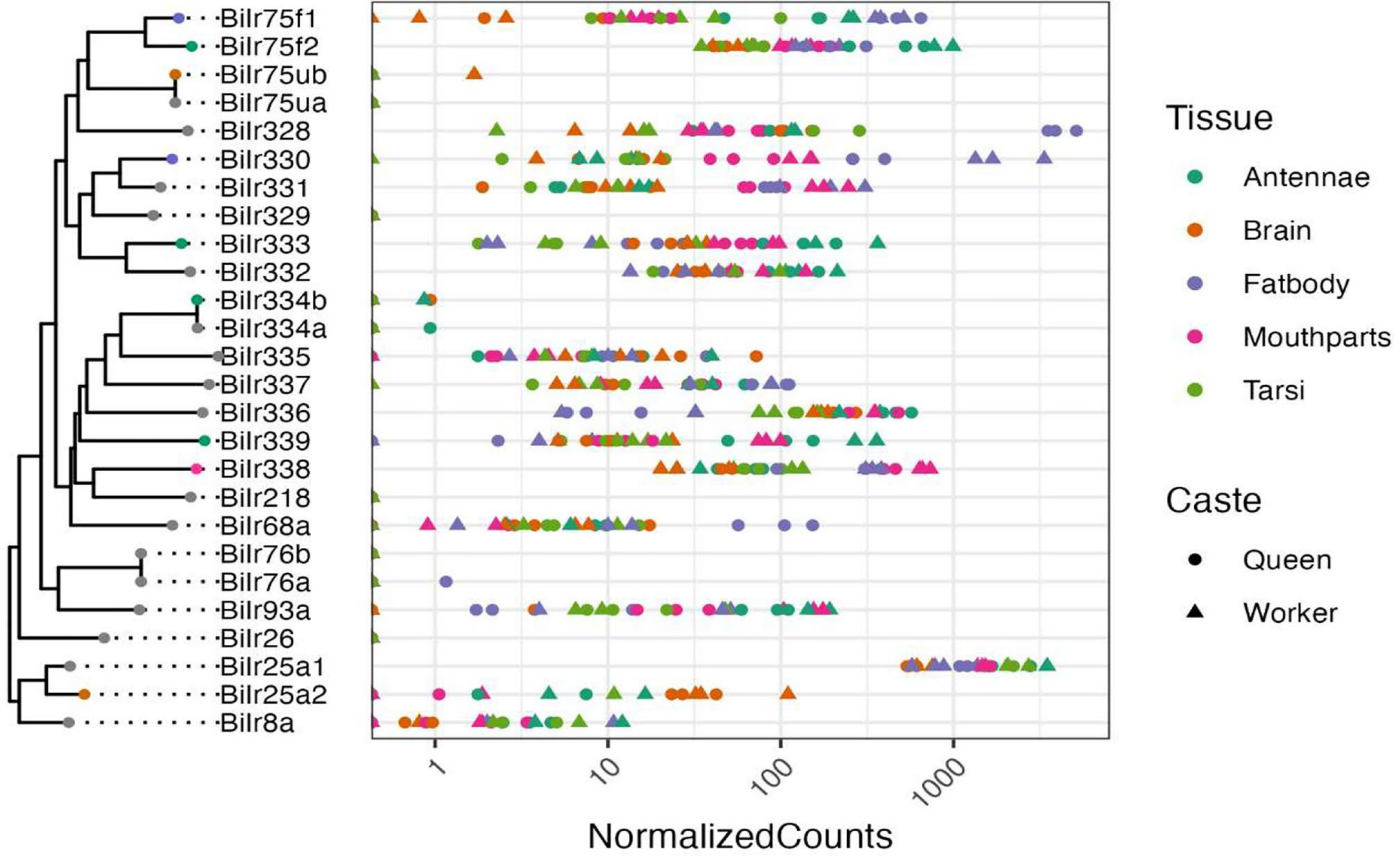
Gene tree with phylogenetic relationships between ionotropic receptors. Gene names with both a and b represent isoforms. Colored circles on nodes of the phylogeny indicate the tissue that each gene is primarily expressed in (τ > 0.8) for the worker caste (queen tissue specificity is not shown). Grey circles on gene tree indicate genes that were not primarily expressed in any tissue (τ < 0.8). Normalized counts of gene expression for each gene represented on the gene tree for each tissue and caste are shown in the graph. The substitution model selected by ModelFinder was LG + F + R3

*Ir* gene expression was the least tissue-specific of all the chemoreceptor gene families, with only 10 of the 26 transcripts (including isoforms) in queens and 9 of 26 in workers showing tissue specificity (Fig. 3; S2). Moreover, there was not a single tissue that had a high number of tissue-specific *Ir* genes. There were, however, more caste-related differences in tissue specificity of *Ir* genes compared to the *Gr* and *Or* gene families. *BiIr25a2* expression was specific to the brain in both queens and workers. *BiIr93a* was specifically expressed in queen antennae but not in any worker tissue. Similarly, *BiIr76a* and *BiIr68a* were specifically expressed in queen fat body but not specifically expressed in any worker tissue. *BiIr338* was specifically expressed in worker mouthparts but not any queen tissue. *BiIr330* and *BiIr75f1* were both specific to the fat body in queens and workers. *BiIr333* and *BiIr339* were both specifically expressed in both queen and worker antennae. Different *BiIr334* isoforms were specific in both queens and workers.

**Fig. 4:**
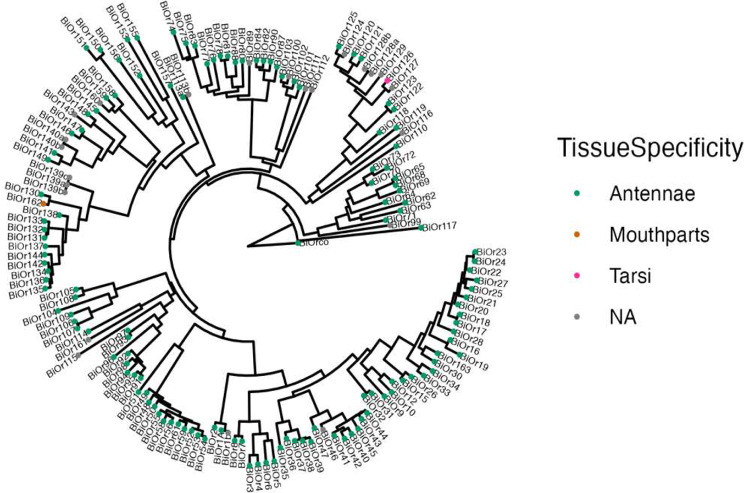
Gene tree with phylogenetic relationships between odorant receptors. Colored circles on nodes of the phylogeny indicate the tissue that each gene is primarily expressed in (τ > 0.8) for the worker caste (queen tissue specificity is not shown). Grey circles indicate genes that were not primarily expressed in any tissue (τ < 0.8). The substitution model selected by ModelFinder was JTT + F + R6

The majority of *Or* genes were specifically expressed in antennae for both queens and workers, which is consistent with their known roles in the detection of volatile compounds [41] (Figs. 4; S3). 128 and 133 genes were expressed specifically in the antennae for queens and workers, respectively. There were a few *Or* genes that were more broadly expressed across tissues (Fig. 4) as well as several *Or* genes that were specific to tissues other than antennae (*BiOr162*: specific to mouthparts in both queens and workers; *BiOr42*: specific to queen brains; *BiOr126*: specific to tarsi in workers).

### General caste and tissue chemoreceptor gene expression patterns

We detected differentially expressed genes in all the chemoreceptor gene families when we compared expression levels across tissues (Figure S4; Supplementary File 3). Our transcriptome dataset included gene expression counts for 15,619 *B. impatiens* genes. 560 (3.6%) of the genes in our dataset are differentially expressed between tissue and caste samples (at an FDR-corrected *p* < 0.05). The majority of differentially expressed genes were *Or*s, whose expression differed the most between antennae and other tissues.

**Fig. 5:**
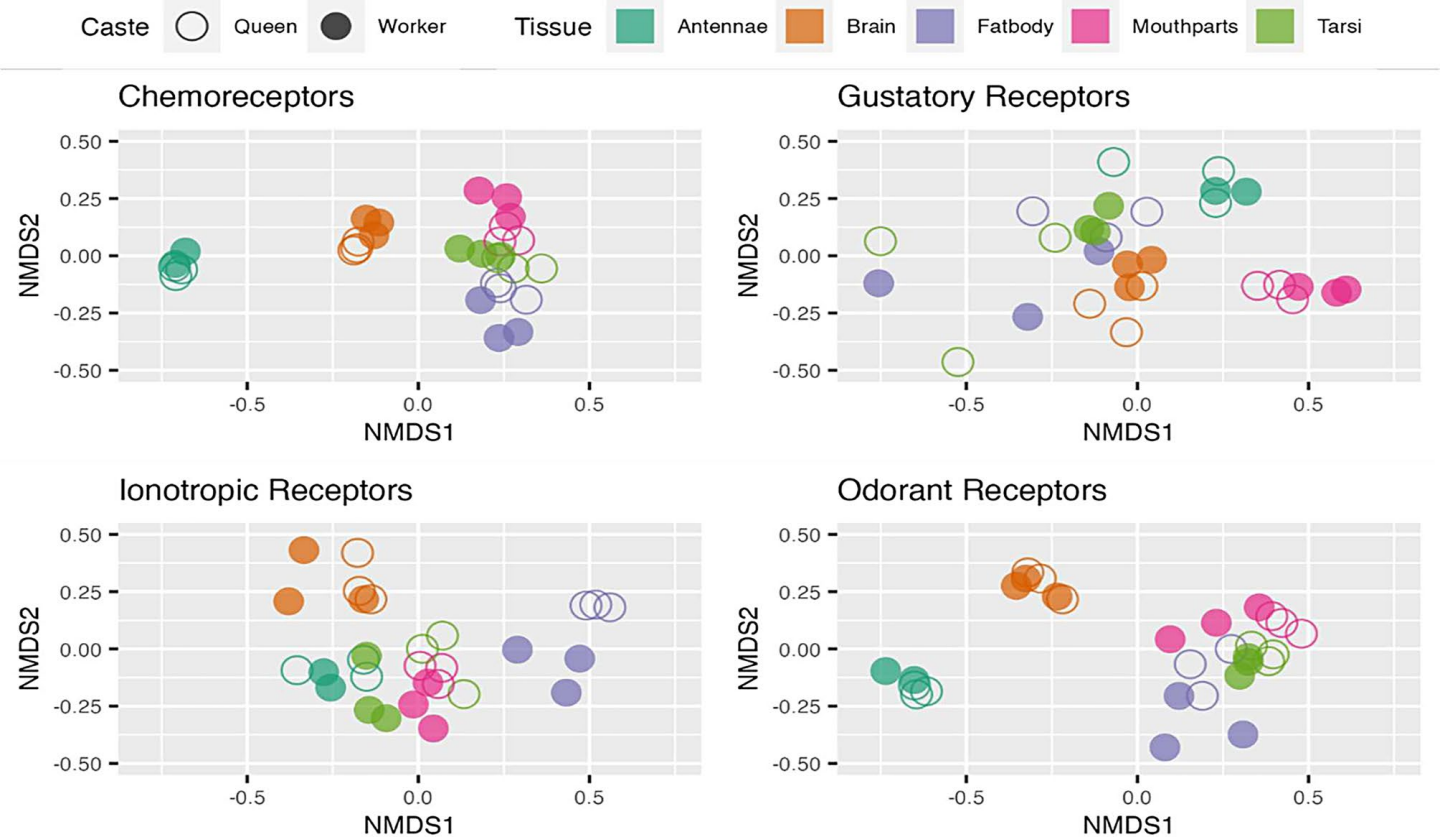
NMDS plots showing similarity in normalized counts of gene expression patterns across tissues and castes for all chemoreceptors, odorant receptors, gustatory receptors, and ionotropic receptors

We performed a non-metric multidimensional scaling analysis (NMDS) to visually compare chemoreceptor expression patterns across tissues and castes. When we considered the 500 most variable genes in the dataset, all samples clustered based on tissue and caste. When the normalized counts of all chemoreceptors were considered, antennal samples formed one distinct cluster, brain samples formed another distinct cluster, and the remaining tissues were the most similar to other samples in their respective groups, but were not as distinct as brain or antennae (Fig. 5). When only *Or*s were analyzed, the patterns were comparable to when all chemoreceptors are considered. The majority of *Or* genes were expressed in the antennae and at substantially higher levels compared to other tissues (FDR-corrected *p* < 0.05; mean log2foldchange = 7.2 +/- 0.18; Figures S4). When only *Gr*s or *Ir*s were considered, there were markedly different patterns. The most distinct groups were the antennae and mouthparts for *Gr*s and the fat body for *Ir*s. There was greater variation in *Gr* expression within replicates for queen tarsi and worker fat body than was detected for other chemoreceptors.

**Fig. 6:**
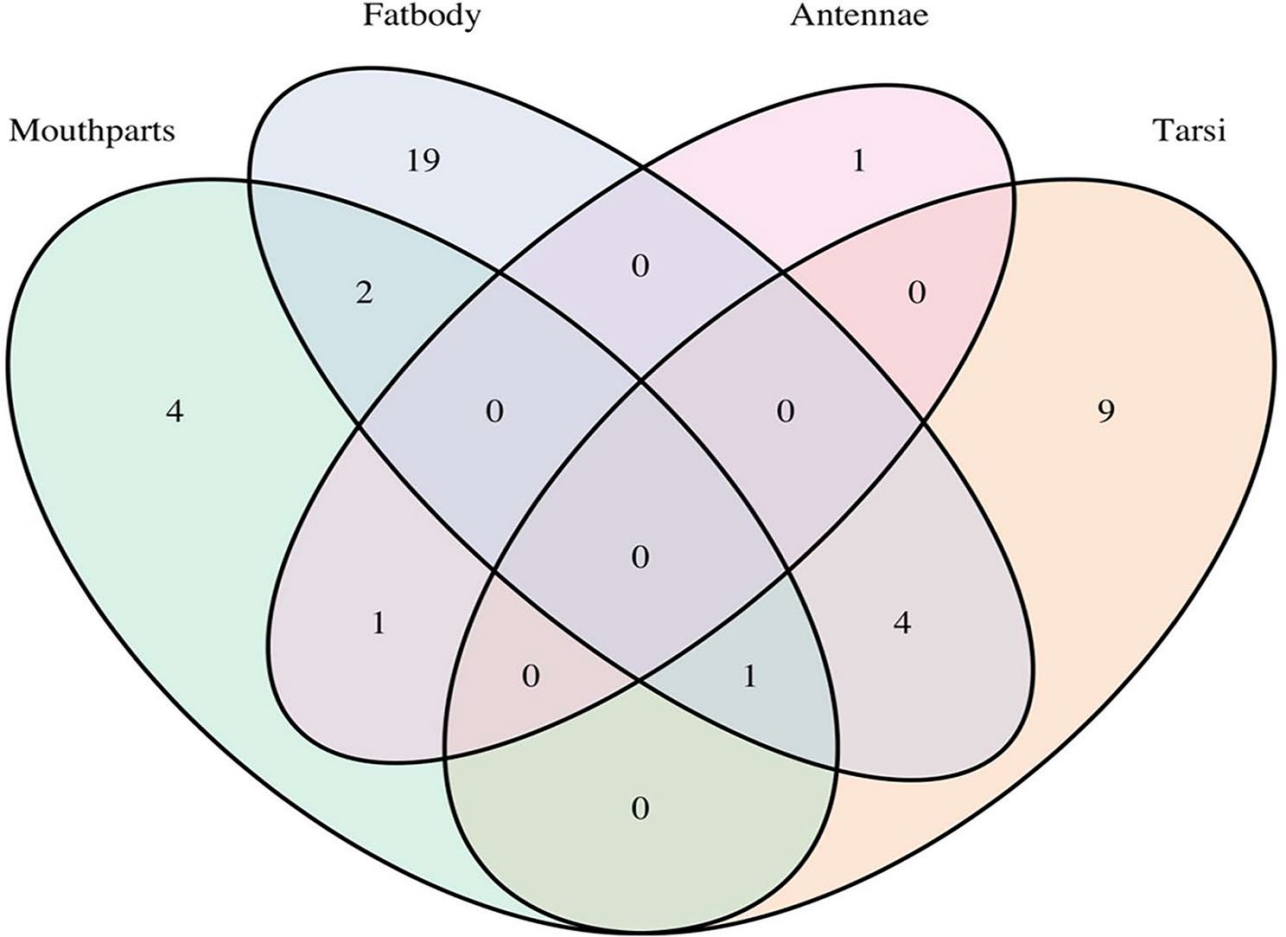
Venn diagram showing the number of shared differentially expressed genes when comparing queens and workers for each tissue

We detected evidence of differential expression between workers and queens in 41 of the chemoreceptor genes (5 *Gr*s, 9 *Ir*s, 27 *Or*s) in the dataset (Fig. 6; Supplementary File 4). Most of these genes were differentially expressed in the fat body (3 *Gr*s, 7 *Ir*s, and 17 *Or*s).

## Discussion

Recently duplicated and species-specific genes can mediate behavioral and ecological shifts, making them intricately linked to evolutionary processes such as adaptation and diversification [1;16;42]. We found that the bumble bee *B. impatiens* has ten recently duplicated *Gr* genes, nine of which are in the three previously identified *Bombus* expansions, and one which is a single duplication from a gene that has simple orthologs in *A. mellifera* and *M. quadrifasciata*. These genes are more likely to be expressed in a subset of sensory organs compared to more conserved *Gr* genes, which were more broadly expressed. This finding provides support for the hypothesis that younger genes have non-essential functions and can subsequently become more generalized over time [20, 43].

Outside of a few insect model systems, relatively little is known about the evolution and function of species-specific chemoreceptor genes in insects. These genes often result from gene duplication, followed by sequence substitutions. This can change receptor properties, altering the compounds the receptors bind to [16, 44] and potentially alter tuning breadth. A study in orchid bees, a lineage in the same subfamily as bumble bees (divergence time between lineages: 60–80 MYA), that compared the tuning properties of two duplicated *Or* genes in a pair of sibling species, for example, found that the more derived variant was more narrowly tuned (Brand et al., 2020). We found that recently duplicated and species-specific genes are more likely to be tissue specific in *B. impatiens*, which may indicate that these genes have less essential and species-specific functions. Moreover, several of these genes were missing the gustatory receptor domains, which can further alter the function of the receptor, which has also been found in other species [14]. This offers additional support that they may have unique functions compared to the ancestral genes they evolved from [45].

The species-specific bumble bee genes were more likely to be specifically expressed in either mouthparts or antennae, compared to *Gr* genes that share orthologs with *M. quadrifasciata* and *A. mellifera*, or more widely conserved *Gr* genes across insects, like the putative sugar receptors. The recently duplicated *Gr* genes in each of the three expansions also followed similar patterns to one another, regarding the tissue they were primarily expressed in, as well as their location in the genome. One expansion included four genes (*Gr12*, *Gr19*, *Gr20*, and *Gr21*) that were all located close together in the genome and all were specifically expressed in the mouthparts. None of the genes in this expansion seemed to share an ortholog with * A. mellifera*. This expansion appears to be from an ancestral gene that is shared with the stingless bee expansion, inferred from *M. quadrifasciata.* The *(B) impatiens* expansion appears to be separate from the *M. quadrifasciata* expansion however, as individual genes in each expansion do not share substantial homology. The other two expansions, (*Gr9*, *Gr17*, and *Gr18*; *Gr8*, *Gr14*, *Gr15*, and *Gr16*) were all located close together in a different part of the genome than the first expansion, except for *Gr16*, and were all expressed specifically in the antennae. *Gr18* and *Gr8* both share orthologs with *(A) mellifera* and *M. quadrifasciata*. Genes that share sequence similarity and are clustered in the same area of the genome often undergo similar evolutionary processes (e.g., types of selection) [46]. Thus, the *Gr12* expansion and the other two expansions that are more distantly located in the genome may be shaped by different selective pressures related to behaviors that the antennae and mouthparts are involved in. On the other hand, as bumble bees interact with food resources and nest materials with both organs simultaneously, it is possible that receptors in each of these organs work in tandem to detect similar compounds in the environment and subsequently shape a single behavioral output. *BiGr22*, the remaining recently duplicated *Gr* gene, is not found in *B. terrestris*, but is duplicated from *BiGr5*, which shares a single ortholog with *B. terrestris*, *M. quadrifasciata*, and *A. mellifera. Gr5* is most closely related to a group of recently identified *A. mellifera* pseudogenes. *BiGr22* may have evolved in a common ancestor of *(A) mellifera* and *Bombus* and been pseudogenized in *Apis* and lost in *M. quadrifasciata* and *(B) terrestris*, or duplicated in *B. impatiens* after diverging from other species in Bombini. *B. terrestris* has one more *Gr* gene compared to *B. impatiens*, but these genes are in the expansions. This result highlights how differences in gene number can be observed even among closely related species, in this case two species in Bombini (estimated divergence between *B. impatiens* and *B. terrestris* = 15–25 MYA [47]).

Our results that *Gr*s are primarily expressed in antennae, in addition to the mouthparts, complements comparative bee morphology and behavior by supporting the idea that antennae are major gustatory organs in bees. This result contrasts with what is known in flies, whose antennae are not typically considered to be major gustatory organs [44]. Some *Gr*s, however, are co-expressed in olfactory organs (e.g., antennae) in flies [48, 49] and a recent study demonstrated that gustatory receptor neurons can be activated by a suite of volatile compounds [49], in addition to carbon dioxide [50]. Bees and other social hymenopterans have much longer antennae than flies [51] and they are geniculate in form. This allows them to contact chemical cues more directly in their environment, such as chemicals on floral resources or conspecific mates and/or nestmates [12]. Given that we found that most *Gr* genes, including recently duplicated ones, are specifically expressed in the *B. impatiens* antennae, gustation may play a more substantial role in mediating behaviors like social communication and floral resource detection, which were previously thought to be shaped by olfaction [13]. Further examining these species-specific bumble bee Grs may help elucidate the functional role of *Gr*s in the detection of volatile compounds, in addition to dissolved compounds, in insects more broadly.

All genes in the chemoreceptor gene families encode receptors that generally function to detect chemicals in the environment. These receptors can be broadly tuned, whereby they detect a wide breadth of chemical compounds, or more narrowly tuned, and detect unique subsets of chemicals [26]. In insects, each chemoreceptor gene family appears to have maintained several highly conserved genes that serve fundamental chemosensory functions, such as serving as co-receptors [7, 25] or as sugar receptors [16]. These orthologous genes appear to maintain similar functions across insects. For example, co-receptors include Orco, which combines with an OrX tuning receptor in olfactory receptor neurons, which are primarily housed in the antennae [41, 52], and the Ir co-receptors (*Ir25a*, *Ir8a*, and *Ir76b*; [53, 54]), which form heterodimers with tuning Irs.

We found the orthologous *Orco* gene in *B. impatiens*, as well as the majority of *Or*s, to be specific to the antennae, which is to be expected based on their role in the detection of volatile compounds associated with conspecifics, nest sites, and floral resources [13]. Our results also match chemoreceptor gene expression patterns in *D. melanogaster*, in which the antennae form a distinct gene expression module, driven by *Or* gene expression, compared to other tissues [21]. The *B. impatiens* orthologs to *Ir25a*, *Ir8a*, and *Ir76b* were all broadly expressed across tissues, which may be due to their role as co-receptors. Fewer *Ir* genes were specifically expressed in any organ, though the few that were, were specific to the antennae. *Ir*s evolved from the ionotropic glutamate receptors and have numerous sensory functions. As tissue specificity is likely indicative of breadth of function, these results indicate that *Ir*s are likely serving multiple sensory functions in *B. impatiens*, which is consistent with what is found in other insects [44, 55].

Our results that the same receptors were expressed both internally and peripherally suggest that individual receptors may receive chemical cues from different locations, and the information is subsequently integrated in the central nervous system to regulate behavior. Receptors expressed in peripheral tissues like the antennae, tarsi, and mouthparts, are likely involved in detecting and discriminating between compounds that mediate nestmate and mate recognition, nest selection and floral resource collection [12, 13, 56], whereas those expressed in internal tissues may be involved in internal nutrient sensing [35, 57]. Chemoreceptors expressed in different organs may evoke different behavioral responses, as they project to different places of integration in the brain [58]. For example, sweet receptors in *D. melanogaster* are also found in different tissues; the detection of compounds by internal and peripheral sweet receptors are integrated in the central nervous system and control different aspects of feeding behavior [59]. We found that both putative sugar receptors in *B. impatiens* were broadly expressed, in addition to others like *Gr18*. This finding suggests that these receptors may have roles in both peripheral and internal chemical detections across multiple behavioral contexts. Peripheral expression of sugar receptors may be critical to ensure they consume enough sugar to meet their physiological needs. There is evidence that fundamental internal nutritional pathways that are highly conserved across insects, such as the insulin signaling pathway, have been coopted to regulate social behavior across multiple social insect lineages [60, 61]. Thus, internal expression of sugar receptors may play a role in these nutritional pathways.

Our finding that some chemoreceptors are also expressed differently between workers and queens suggests that bumble bee caste differences extend to chemical perception, although the specific details of how they differ remain to be understood. Expression differences between castes in the peripheral tissues may be related to behavioral differences pertinent to nestmate communication and feeding and foraging behavior. For example, differences in chemoreceptor expression may alter the sensitivity of workers to signals related to nest defense and reproductive dominance compared to queens. However, more functional ecology studies will be needed to test this. Similarly, some of these differences may be related to food collection and feeding behaviors. Genes that are differentially expressed in the fat body may be related to differences in nutritional pathways that are involved in reproductive division of labor. A limitation of our study is that we compared workers in the nest to unmated new queens in the nest (gynes). There are widespread differences in queen and worker physiology; for example, queens live approximately one year, undergo diapause and reproduce, whereas workers only live several months and do not undergo any drastic physiological changes like diapause or reproduction. Moreover, the sensory related behaviors that queens perform vary substantially between these life history stages. Thus, comparing gene expression differences between workers and queens at different life history stages (e.g., solitary, nest-founding queens performing feeding and foraging activities, gynes, and social queens) may better contextualize these patterns.

## Conclusion

Chemoreception mediates fundamental behaviors in bumble bees like finding mates, selecting suitable nest sites, and foraging resources [12, 13]. Despite this, the molecular basis of bumble bee chemoreception, and the importance of each chemoreceptor family to bumble bee behavior and ecology, are still relatively uncharacterized. Nearly all functional studies on chemoreceptors in bees have been carried out in the western honey bee *A. mellifera* and involve in vivo electrophysiology recordings from chemosensory sensilla or heterologous expression systems such as the *D. melanogaster* neuron system and *Xenopus* oocytes [57]. Yet still, much of what is known about the function of individual chemoreceptor genes in bees is inferred based on homology with genes in *D. melanogaster*, where more functional studies have been performed. Our study provides a foundation for determining which tissues to target for functional studies for gustatory, ionotropic, and olfactory receptors in *B. impatiens*.

## Electronic supplementary material

Below is the link to the electronic supplementary material.

Supplementary Material 1

Supplementary Material 2

Supplementary Material 3

Supplementary Material 4

Supplementary Material 5

Supplementary Material 6

Supplementary Material 7

Supplementary Material 8

## Data Availability

The transcriptomic datasets generated during the current study are available in the National Center for Biotechnology Information database (Bioproject: PRJNA1150589). All other associated data and data analysis scripts are available on open science framework (https://osf.io/z7htk/?view_only=ff6dac47283641ca920e70d6e78a614e).
