# Supplementary figures and images for: Expression analyses of chemosensory genes provide insights into evolution of gustatory receptor genes in the bumble bee Bombus impatiens
Source: BMC Genomics. 2025 Jul 1;26:575. doi: 10.1186/s12864-025-11710-x (PMC12211280; doi:10.1186/s12864-025-11710-x)

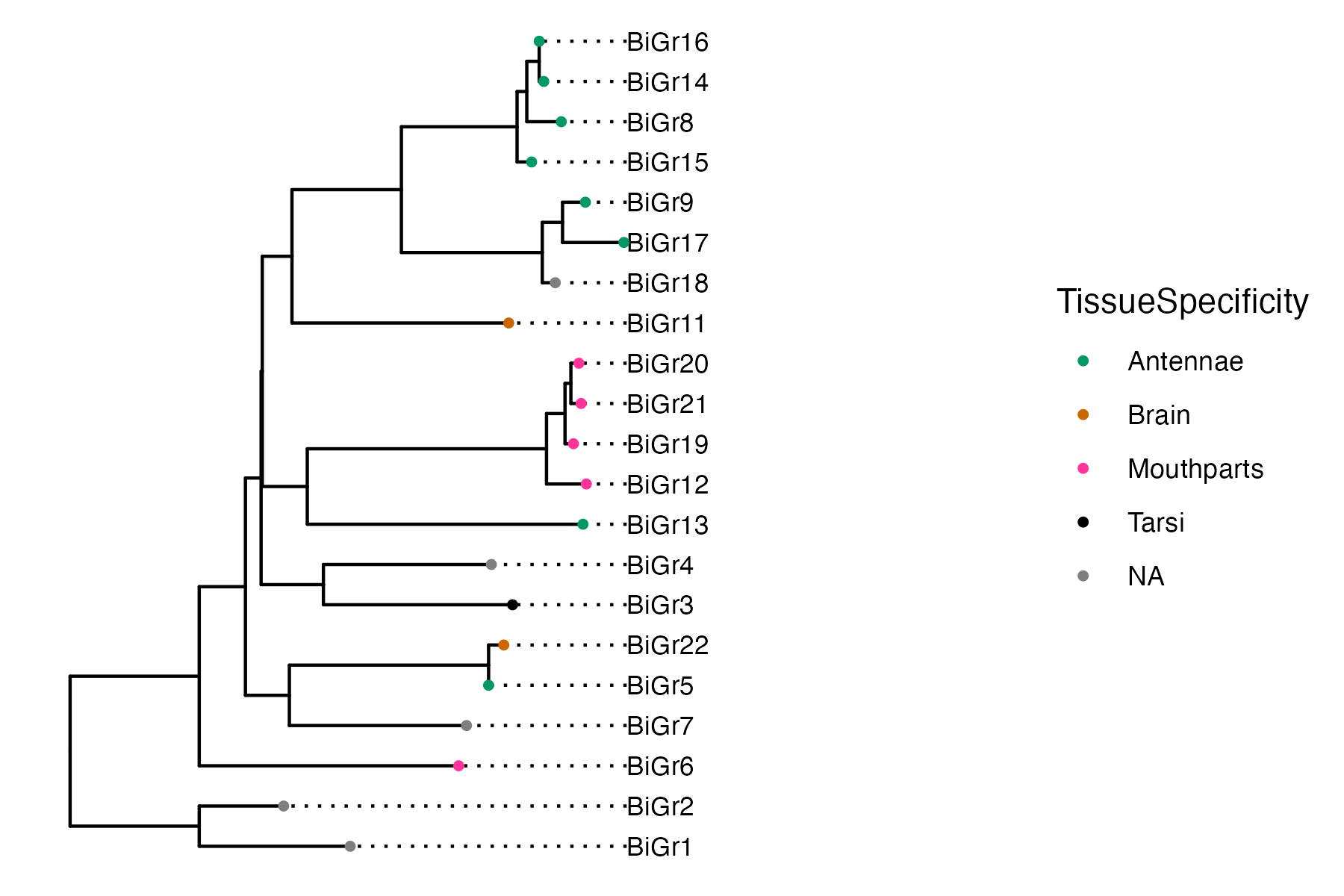

Supplement: Supplementary file 1 — Supplementary Material 1 [file 12864_2025_11710_MOESM1_ESM.png]

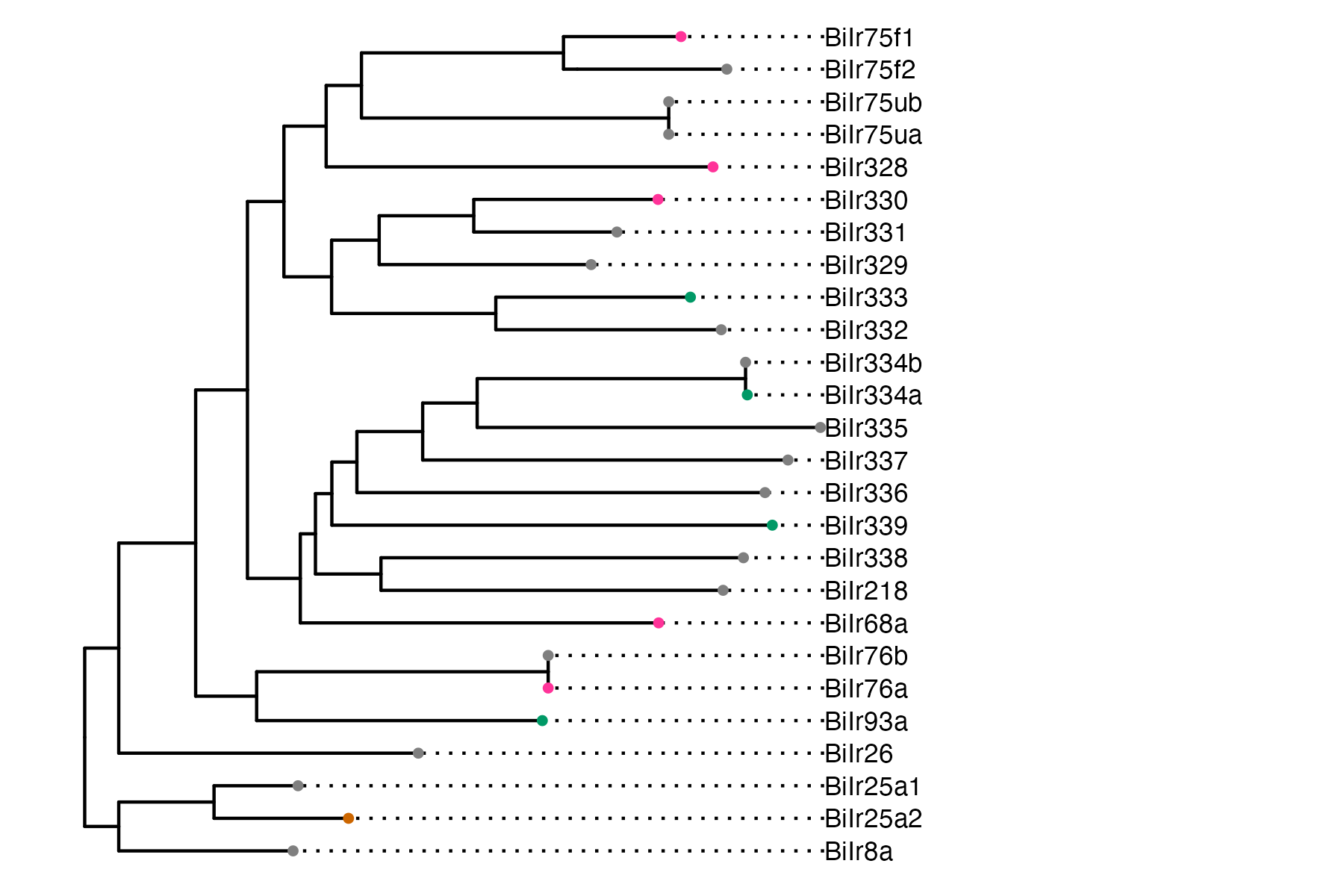

Supplement: Supplementary file 2 — Supplementary Material 2 [file 12864_2025_11710_MOESM2_ESM.png]

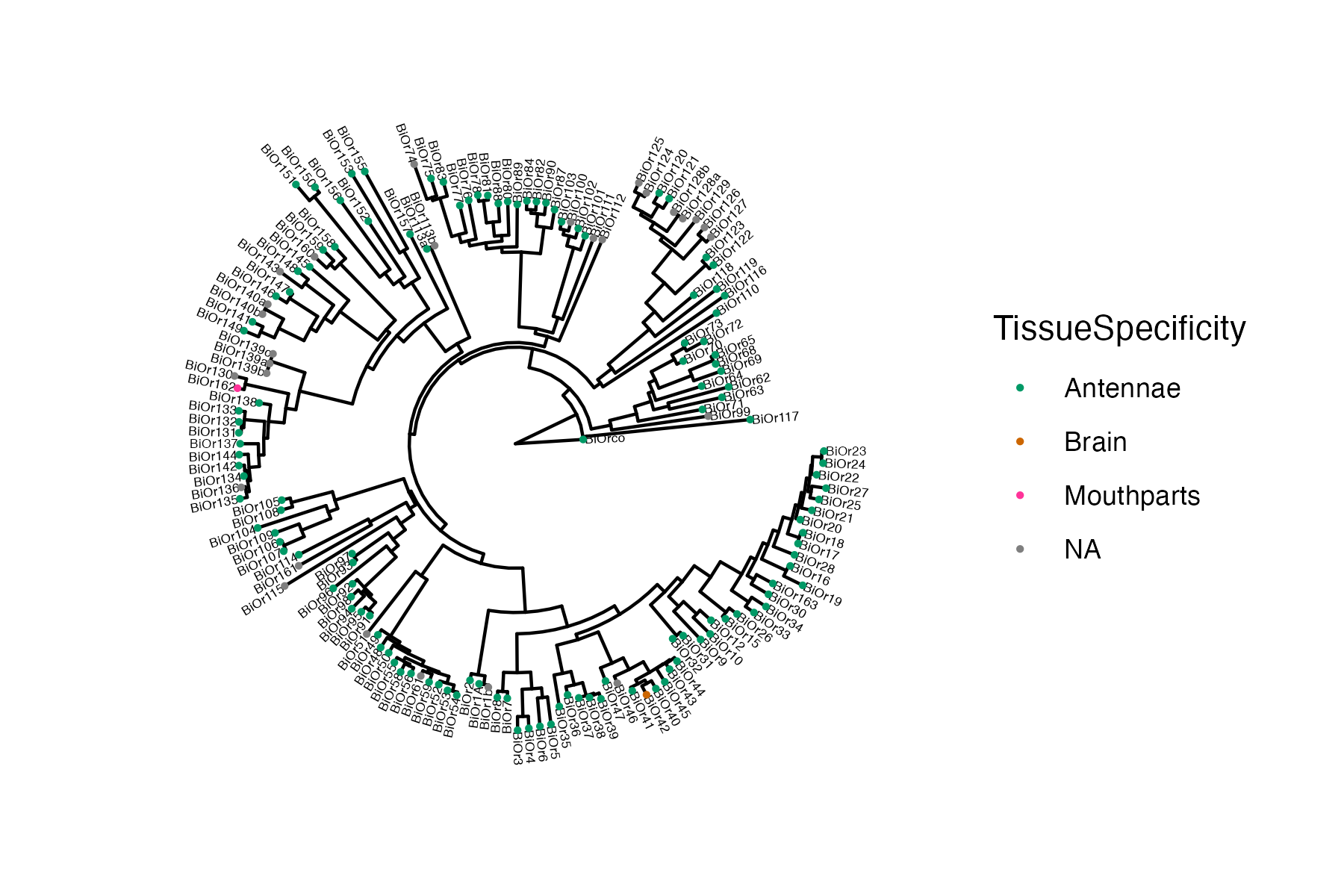

Supplement: Supplementary file 7 — Supplementary Material 7 [file 12864_2025_11710_MOESM7_ESM.png]

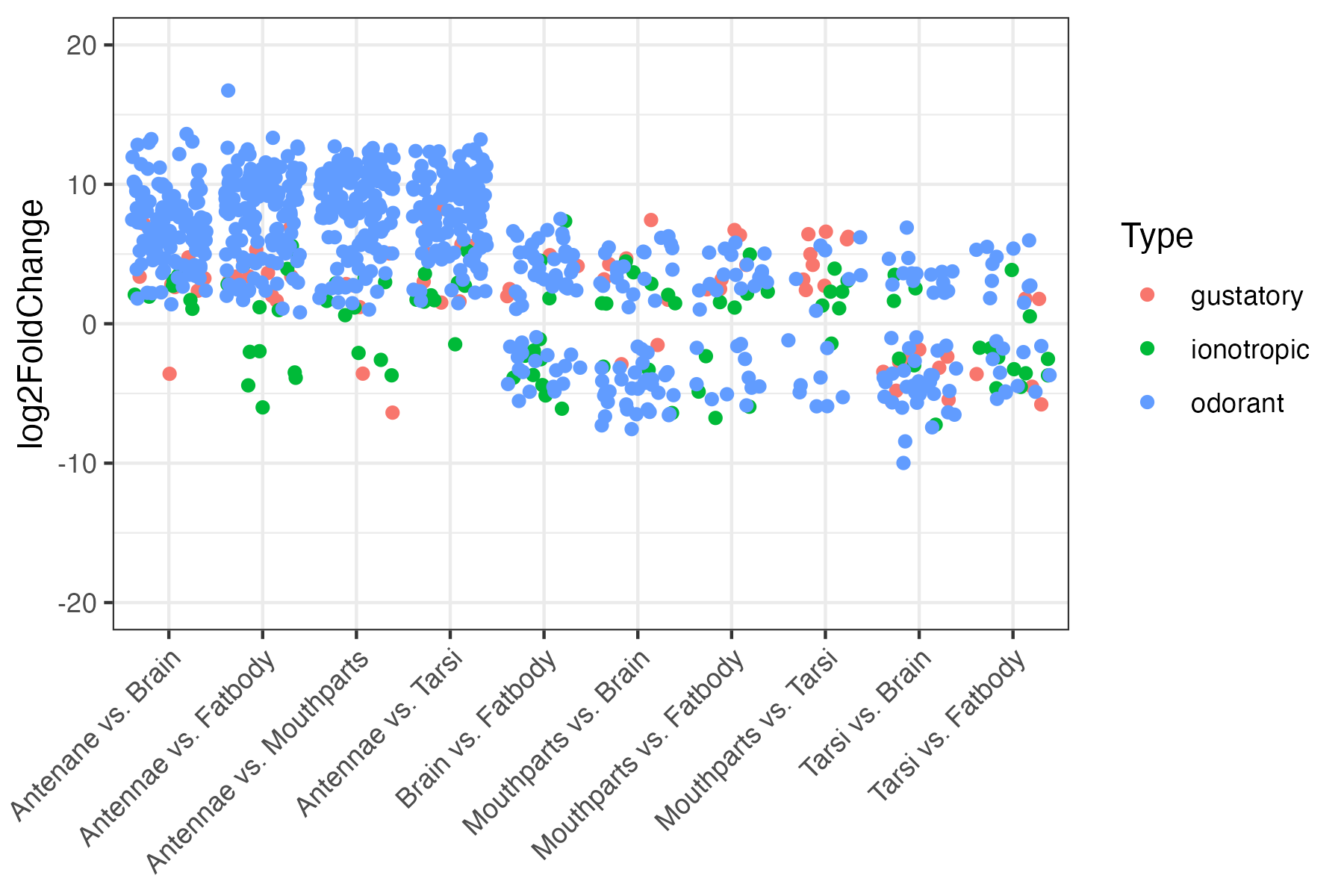

Supplement: Supplementary file 8 — Supplementary Material 8 [file 12864_2025_11710_MOESM8_ESM.png]
